# Biomarkers in Prostate Cancer Epidemiology

**DOI:** 10.3390/cancers3043773

**Published:** 2011-09-30

**Authors:** Mukesh Verma, Payal Patel, Mudit Verma

**Affiliations:** 1 Epidemiology and Genetics Research Program, Division of Cancer Control and Population Sciences, National Cancer Institute, National Institues of Health (NIH), 6130 Executive Blvd., Rockville, MD 20852, USA; E-Mail: pvpatel@umd.edu; 2 Laboratory of Cancer Biology and Genetics, Clinical Research Center, National Cancer Institute, National Institues of Health (NIH), 9000 Rockville Pike, Bethesda, MD 20892, USA; E-Mail: mverma@umd.edu

**Keywords:** biomarker, cancer detection, diagnosis, epigenetics, genetics, prostate cancer, treatment

## Abstract

Understanding the etiology of a disease such as prostate cancer may help in identifying populations at high risk, timely intervention of the disease, and proper treatment. Biomarkers, along with exposure history and clinical data, are useful tools to achieve these goals. Individual risk and population incidence of prostate cancer result from the intervention of genetic susceptibility and exposure. Biochemical, epigenetic, genetic, and imaging biomarkers are used to identify people at high risk for developing prostate cancer. In cancer epidemiology, epigenetic biomarkers offer advantages over other types of biomarkers because they are expressed against a person's genetic background and environmental exposure, and because abnormal events occur early in cancer development, which includes several epigenetic alterations in cancer cells. This article describes different biomarkers that have potential use in studying the epidemiology of prostate cancer. We also discuss the characteristics of an ideal biomarker for prostate cancer, and technologies utilized for biomarker assays. Among epigenetic biomarkers, most reports indicate GSTP1 hypermethylation as the diagnostic marker for prostate cancer; however, NKX2-5, CLSTN1, SPOCK2, SLC16A12, DPYS, and NSE1 also have been reported to be regulated by methylation mechanisms in prostate cancer. Current challenges in utilization of biomarkers in prostate cancer diagnosis and epidemiologic studies and potential solutions also are discussed.

## 1. Introduction: Prostate Cancer Incidence and Prevalence

Prostate cancer is the second most frequently diagnosed cancer as well as the sixth leading cause of death in males with cancer worldwide [1-18]. In the United States alone, prostate cancer is the most common cancer after skin cancer and is the second leading cause of cancer death in men [19]. Overall, the incidence rate is 156 per 100,000 men per year for all races, and one in six men in the United States is affected by prostate cancer [20]. The number of estimated new cases of prostate cancer in males is about 903,500, and the estimated number of deaths in this population is 258,400 [21]. In 2008, in the United States alone, more than 2 million men were alive who either once had prostate cancer or who had it at that time [3]. Although only up to 10% of patients actually die from this disease, there is extensive information in the fields of biology and epidemiology that remain unknown to scientists [22].

**Figure 1.**
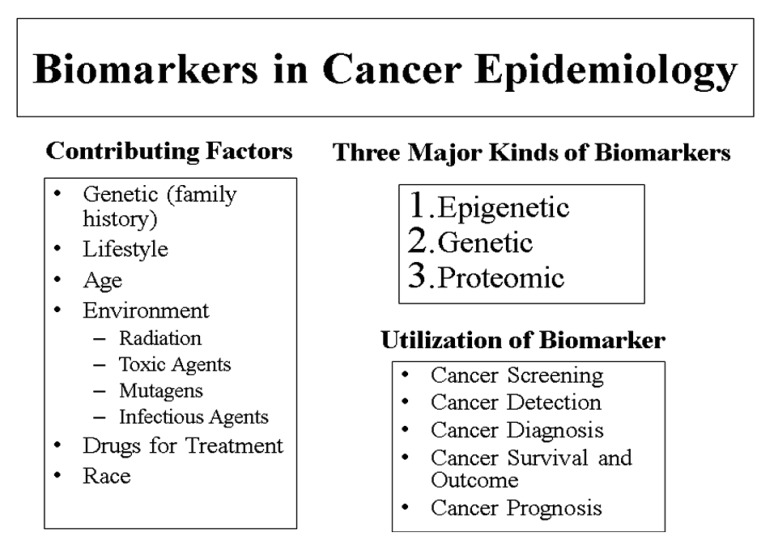
Major risk factors of prostate cancer and a broad classification of biomarkers of prostate cancer.

Several risk factors for prostate cancer have been identified. Although no preventable risk factors have been determined, the most common identified risk factors include old age, race, and family history (Figure 1). More specifically, those at higher risk for developing prostate cancer include men over the age of 65, African American (AA) men, and men with brothers or fathers who have had prostate cancer [20]. In addition, men who abuse alcohol, maintain diets high in fat, and have been exposed to cadmium or Agent Orange also are at risk for prostate cancer. Men who work in tire plants or mines or who are painters may be at risk as well [20]. Several studies have found that males of African descent have the highest prostate cancer incidence and mortality rates, whereas native Asians are least likely to develop this type of cancer [21]. Nonetheless, prostate cancer incidence and mortality rates are increasing in some Asian and European countries [3].

## 2. The Importance of Understanding Prostate Cancer Biology and Epidemiology

One of the largest uncertainties in this field is the actual origin of prostate cancer. Many risk factors have been identified for potential sufferers, but the exact causes have yet to be discovered [21]. In addition, diagnosis of prostate cancer in its early stages is difficult. Specifically for Stage I patients, the cancer cannot be seen on a sonogram or diagnosed during a rectal exam. Stage II prostate cancer also can remain unidentified during a rectal exam or sonogram [23]. Ductal prostate cancer is a rare histological variant of prostate cancer. The incidence, natural history and outcomes of patients with ductal prostate cancer have not been identified extensively. Despite a stable incidence, ductal prostate cancer remains an aggressive prostate cancer variant usually presenting with advanced clinical stage and resulting in a high rate of prostate cancer specific mortality.

Prostate cancer takes different amounts of time, sometimes up to several years, to metastasize and the time of cancer development varies in different patients. Some cases may be more severe than others, and some prostate cancers have slower courses that may not lead to immediate clinical symptoms [23]. Prostate cancer has the ability to metastasize to lymph nodes and bones, which is a growing concern for doctors and patients. In addition, there is no consensus on treatment, and the best treatments for various cases of this cancer are not always clear to physicians. On several occasions, complications of prostate cancer resulting from different treatments have been reported. The treatment of prostate cancer is costly. The majority of men are treated with radiation, surgery and chemotherapy, but even watchful waiting strategies are expensive. With increasing life expectancy a large number of men are being diagnosed with this disease, thus effectively increasing the economic burden of the disease.

To understand the biology of prostate cancer, we have to appreciate the role of the androgen receptor signaling in the development, function, and homeostasis of the prostate. Androgen receptor regulates gene transcriptional process by nuclear translocation, binding of receptor to androgen response element on target genes and crosstalk with transcription factors. For advanced prostate cancer treatment androgen deprivation therapy is norm. However, majority of patients progress to a more aggressive, castrate-resistant phenotype. Understanding the underlying process and complexity of androgen receptor signaling in the progression of castrate-resistant prostate cancer is essential for developing successful therapies of this complex cancer type. Furthermore, research in the second-line setting of castrate-resistant phenotype prostate cancer to optimize treatment options, with the objectives of survival prolongation, improvement in quality and pain management also is needed.

Chinnayian's group has identified androgen regulated prostate-specific serine protease (TMPRSS2) and ERG gene fusions, TMPRSS2-ERG, as the predominant molecular subtype of prostate cancer and demonstrated that TMPRSS2-ERG fusions help in distinguishing between PIN and prostate cancer [24-26]. The discovery of genes fused together is a major advancement in the understanding of prostate cancer. Research in Chinnaiyan's group is about using these “gene fusions” to identify prognostic categories to improve approaches to the treatment of prostate cancer patients. Previously it was reported that the majority of prostate cancers have a chromosomal rearrangement as a result of fusion of gene encoding TMPRSS2 with transcription factor ERG [27]. Fusion products can be identified by RT-PCR, expression profiling using exon array or FISH. Presence of specific fusion products is correlated with poor survival. Recent data demonstrates that progression of prostate cancer involves interaction of PTEN and phosphoinosotide-3-kinase pathway and few biomarkers have been identified which may be therapeutic target of prostate cancer [28]. Involvement of repressive epigenetic programs via a Polycomb group of proteins, H3K27 methyl transferase EZH2, also has been demonstrated, which helps us understand the mechanism underlying progression of prostate cancer [29]. A number of somatic mutations and alterations in gene copy numbers associated with aggression and lethality of prostate cancer also have been identified [30,31].

In terms of epidemiology, researchers have observed a correlation between racial background and development of prostate cancer. AA men are at the greatest risk of developing prostate cancer, whereas Asian men living in Asia have the lowest risk [32]. However, this risk increases if an Asian man moves to a Western country such as the United States. Specific reasons and mechanisms behind why these varying levels of risk exist and change have not yet been established, but research is being conducted in this area [23]. Genome-wide association studies (GWAS) have identified several single nucleotide polymorphisms (SNPs) that are independently associated with small increment in risk of prostate cancer suggesting the possibility for using such variants in risk prediction.

Factors contributing to prostate cancer, such as lifestyle, environment, exercise, tobacco use, radiation, exposure to pesticides, infectious agents, have been identified in few epidemiologic studies [31,33]. The effects of salt and processed meat, consumption of filtered and boiled coffee and their effect on prostate cancer incidence rate also have been studied. For prevention of aggressive prostate cancer, consumption of vegetables, fruits, grains, and high glycemic index foods was observed to be beneficial in a case-control study involving about 1,000 participants [34]. Selenium is also a good candidate for prostate cancer prevention [35]. Other lifestyle activities which may reduce the risk of prostate cancer are a healthy diet and weight management, regular exercise, reduction in alcohol consumption, and smoking cessation. In addition, links between receiving a diagnosis and treatment for prostate cancer and development of psychosocial disturbance via emotional negativity, decreased cognitive performance and withdrawal from others needs further research. Overall, prostate cancer and its causes, progression, and treatment remain mystery to physicians and researchers. This disease currently affects thousands of men around the world, and the rates of incidence are continuing to increase. The need for a better understanding of the biology and epidemiology of prostate cancer is urgent and necessary to help medical professionals, prostate cancer patients, and men at high risk for developing this disease. Prostate cancer has difficulties in drug development for treatment and patient management because imaging technologies used to assess disease in bone, which is the most common site of spread, has not been standardized and may not reflect the status of the disease accurately. Additionally, the association between a given post-therapy change in PSA and survival is modest and, that it is not appropriately accepted by regulatory agencies for drug approvals [36,37]. Another area of research is related with the heterogeneity of treatment effects and expenditure which directly determine the cost-effectiveness of health interventions. The study objectives should include analysis of the variation in costs, effects and incremental cost-effectiveness ratios associated with treatment in elderly patients with metastatic prostate cancer in different races and ethnicity groups.

## 3. Biomarkers of Prostate Cancer Detection, Progression, Risk Assessment, Prognosis and Survival

Conventionally, prostate cancer is detected by digital rectal examination, histopathological analysis, and prostate specific antigen (PSA) assays [38,39]. However, these techniques have limitations, and new molecular biomarkers are being characterized that potentially help in detecting the disease, risk assessment, and follow-up of treatment and survival [40]. Biomarkers are molecules that serve the purpose of distinguishing abnormal biological processes, such as diseases or cancers from normal processes [41]. These molecules may be proteins, chemicals, or even segments of DNA and RNA [42-44]. To be classified as a biomarker, a molecule must be related to some occurrence such as the diagnosis of a certain disease, progression, or survival for a specific patient. Not all biomarkers are equally effective, but most do provide additional information to what already has been determined by clinical and pathological analyses [42,43].

Several uses for biomarkers have been established [34]. Detection of a disease itself and progression of a disease are important biomarker functions. Additionally, biomarkers are used in predicting outcomes after administering certain treatments and clinical interventions. Other areas in which biomarkers are utilized include risk assessment, diagnosis, and the development of medications [42].

**Figure 2.**
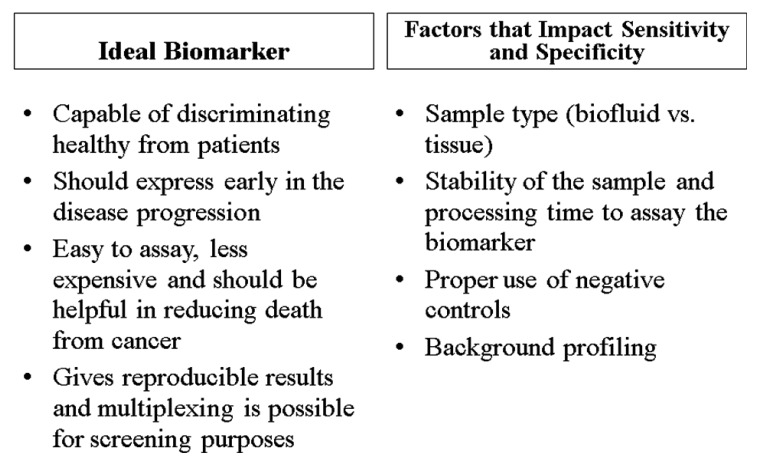
Characteristics of an ideal biomarker and factors influencing sensitivity and specificity of biomarkers.

Ideally, biomarkers have several common characteristics (Figure 2). They are inexpensive, reliable, consistent, easily measured, and their expression is altered under disease conditions. Specifically for prostate cancer, biomarkers should be able to ascertain the presence of the disease, supply information about progression, assess the effectiveness of treatment, and predict the likelihood of recurrence and survival [42]. Many biomarkers may also be able to detect prostate cancer in its premature stages. Overall, biomarkers should have high specificity for the diagnosis and prognosis of a disease and reduce the rate of false-positives [42,44]. The significance of biomarkers in epidemiologic studies relates with their ability to distinguish high risk from low risk patients; patients who should be treated aggressively; and patients who are responding to treatment as opposed to those which do not respond. This means that patients who fall into the category of clinically insignificant disease, based of their biomarker profiles, can be identified with reasonable accuracy and that patients who are initially classified as low risk who reclassify over time as higher risk and are treated radically are still cured in most cases. This may also reduce the psychological burden of living with untreated cancer.

Three common genres can be used to organize prostate cancer biomarkers: predictive, diagnostic, and prognostic (Figure 1). Predictive biomarkers serve to evaluate whether or not a person will develop prostate cancer. Diagnostic biomarkers distinguish cancerous cells from noncancerous cells [39]. Finally, biomarkers are used prognostically to predict the progression and outcome of prostate cancer as well as identifying details about treatment for certain patients [42].

In addition to being designated as predictive, diagnostic, and/or prognostic, prostate cancer biomarkers also can be classified by the type of molecule and mechanism of action involved. These include genetic, epigenetic, and proteomic biomarkers (Figure 1, Table 1). Genetic biomarkers usually are DNA-based and are associated with changes in the DNA itself, such as chromosomal losses, gains, and translocations [44]. Epigenetic biomarkers are associated with changes in DNA that are not involved with the DNA sequence itself. Instead, common epigenetic biomarkers often are related to changes in DNA methylation, histone modifications, microRNA profiles and chromatin modifications [44]. Proteomic biomarkers deal with proteins and specifically for prostate cancer, proteomics deals with finding proteins and patterns of proteins that may be correlated with prostate cancer (Table 1). Each of these biomarkers can have predictive, diagnostic, and prognostic values. Other categories of biomarkers exist as well, although the abovementioned classifications are among the most common. In the biomarker field, nomograms are also used. Nomograms are models that take multiple, disease-specific inputs and use those factors to predict the likelihood of a specific outcome. Namograms are designed to be used by patients who have had a biopsy of the prostate confirming the presence of cancer. The biopsy must have had a Gleason grade assigned to it. In addition, patients will need other clinical data. A combination of disease factors including stage of the cancer, PSA level, biopsy pathology, use of hormone therapy, and radiation dosage are incorporated into the nomograms. For men who have received no primary treatment, the nomogram calculates the statistical probability of a cancer remaining progression-free after receiving one of three treatment options: prostatectomy, external beam radiation therapy, and brachytherapy. For men who have been treated with a prostatectomy, nomogram predicts probability of survival; for men who have experienced a recurrence of their prostate cancer after being treated with a prostatectomy, nomogram predicts treatment success for salvage radiation therapy (SRT); and for men who have received either prostatectomy or radiation therapy and are considering hormone refractory treatment (HRT), this tool predicts one- and two-year survival after HRT. Furthermore, this tool can be used to predict the probability and time to the development of metastatic disease. Additionally, a nomogram calculates prostate volume, which is used to interpret PSA results.

Although we describe individual biomarkers in the following section and Table 1, the specificity and sensitivity of diagnosis is optimum when several biomarkers are used in combination [39]. Before making a decision about disease pathogenesis, a patient's family history and lifestyle should be taken into account. The characteristics of an ideal biomarker and factors that influence the specificity as well as the sensitivity of a biomarker are shown in Figure 2.

Biomarkers have been known for a long time but the approach and methods have changed in recent times, primarily because of the advancement in technologies. Traditionally, scientists have relied on conventional tools, such as gel electrophoresis and immunohistochemistry to follow gene and protein expression. The main problem of this approach was that only a limited number of biomarkers could be identified and studied simultaneously. Furthermore, prior knowledge about biomarkers was required in the traditional approach of using biomarkers in prostate cancer epidemiology. New methods based on high throughput genomic, proteomic, and epigenomic analysis of prostate samples have made it possible to use multiple biomarkers simultaneously. The advantage of modern technologies is to identify alterations in genome, proteome, or epigenome and link them with disease state and/or a response to a medical intervention. Microarrays are used for DNA, RNA or proteins, and bisulfate treated DNA is used for methylation profiling. Mass spectrometry, liquid chromatography, and protein arrays are employed to characterize peptide and protein profiles. Chip-on-Chip assay to determine alterations in histone profiles of prostate samples is routine. Sophisticated algorithms and bioinformatics are applied to determine association of biomarkers with prostate cancer detection and progression. Proper steps and protocols have been developed for analytic and clinical validation of biomarkers [41]. The consensus among scientists indicates that a test used to make clinical decisions must lead to a beneficial impact on patient's outcome. Thus, use as a clinical diagnostic also involves evaluation of benefits, harm cost, and efforts [45]. Recently lots of attention has been paid to isolation of circulating tumor cells (CTC) and disseminated tumor cells (DTC) from blood of prostate cancer patients [46]. An early spread of cells to lymph nodes or bone marrow is referred as circulating tumor cells or as disseminated tumor cells when they are present in the blood [47]. These cells can be enriched by density gradient centrifugation and immunomagnetic procedures [48]. DNA or RNA can be isolated from these cells to identify disease associated biomarkers. Reverse transcriptase PCR and CTC-chip methodologies are applied for further characterization. For routine use of CTCs and DTCs in therapy decision making, randomized prospective trials will be suitable.

### 3.1. Genetic Biomarkers

Many genetic alterations occurring during development and progression of prostate cancer have been identified over the last two decades. Briefly, recurrent common chromosomal aberrations are losses and gains of chromosome 8p and 8q, respectively, losses at 5q, 6q, 10q, 13q, 16q, 18 and gains at 1q, 3q, 7 and Xq12, respectively. It is not likely that there is a single linear sequence of genetic alterations during prostate cancer progression. Rather, these alterations occur as preferred combinations and to different degrees in cancers with different clinical course. However, either alone or in combination they do not allow a sufficiently precise sub-typing for clinical practice. Although most of the above chromosomal losses or gains have been defined for quite some time, which specific genes on each chromosome are associated with prostate cancer is still debated. Allelic loss or mutations of “classical” tumor suppressors, PTEN, RB1 and TP53, are predominantly found in advanced stage prostate cancers and mutations in common protooncogenes, such as the RAS family are rare overall. To identify high risk populations, two approaches, candidate gene and GWAS, have been conducted [49-52]. The overarching goal is to discover the pathways that drive prostate cancer pathogenesis and to assess their role in clinical decision making. These studies have identified prostate cancer associated genetic variants. Although most of these studies have been conducted in populations of European descents, few studies include men of African and Asian descents and men with a family history of prostate cancer [53,54]. More than 30 SNPs have been identified to date and majority of these SNPs are located at the 8q24 region. Results from studies in different populations suggest that genetic etiology of prostate cancer is different in descents of European, American, African, and Asian populations [52,55,56]. Large epidemiological studies are needed to confirm above results. Validation of identified risk-associated SNPs is needed before they can be used for screening purposes. Identification of prostate cancer associated genetic variants may improve our understanding of the disease etiology and have potential implications for the early detection, diagnosis, and treatment of prostate cancer. The focus of some of the projects is to investigate the potential of this new knowledge on the genetic basis of prostate cancer susceptibility to enhance risk assessment, through gene-gene and gene-environment interactions, and importantly, to provide the potential for novel clinical practices through impacts on cancer diagnosis and treatment, or newer cancer prevention strategies.

Genetic biomarkers often are associated with the overexpression of a gene. This is the case for ERG, ETV1, PCA3, GOLPH2, MYC, PIM1, and the gene expressing hepsin as described below.

#### a. TMPRSS2-ERG gene Fusion Rearrangement

ERG and ETV1 are overexpressed in prostate cancer, but they also fuse with TMPRSS2, which leads to tumor progression. The fusion of these genes can be detected in urine, and this TMPRSS2-ERG gene fusion rearrangement may aid in predicting prostate cancer development [42,57-59]. In addition, monitoring gene transcripts of the gene fusion may improve the sensitivity of detection of PCA3, which is another biomarker.

#### b. PCA3

The gene for prostate cancer antigen 3 (PCA3) encodes prostate-specific noncoding mRNA. It is measured in urine and has the potential to enhance the diagnosis of prostate cancer as well as its staging, grading, and aggressiveness. An advantage of using PCA3 as a biomarker is that it has good specificity and can distinguish between prostate cancer and benign conditions, thus improving the detection of this cancer compared to PSA [59,60]. Treatment selection for prostate cancer should be based on a combination of clinical and pathological variables. If one wants to use a threshold point to guide treatment decisions in clinical practice, a PCA3 score threshold of 20 may have the highest utility for selecting men with clinically insignificant prostate cancer in whom active surveillance may be appropriate; a PCA3 score threshold of 50 may be used to identify men at high risk of harboring significant prostate cancer who are candidates for radical prostatectomy.

#### c. GOLPH2

GOLPH2 is a gene coding for Golgi phosphoprotein 2, which is a Golgi membrane antigen. This gene is upregulated in about 90% of prostate cancer cases, leading to overexpression of the gene. GOLPH2 serves as a biomarker in diagnosis and aids in distinguishing between normal and cancerous cells [42]. This marker can be assayed in urine. Up to now urine-based biomarkers represent a promising alternative or addition to serum-based biomarkers. Prospective studies in a multivariate setting, including larger sample sizes and avoiding attribution bias caused by preselection on the basis of serum PSA are however required.

#### d. PIM1

PIM1 is a gene that encodes a protein kinase. Although there is little or no PIM1 expression in the benign prostatic epithelium, there is significant PIM1 expression in advanced cases of prostate cancer. For this reason, PIM1 serves as a prognostic factor [61]. PIM1 has a possible role in other carcinomas with genetic alterations (SNPs and mutations) in 6p21 region. On one hand, PIM1 (due to its role in malignancy) appears to be a promising target for drug development programmes but, on the other hand, the complexity of its molecular structure has given few opportunities for the development of PIM1 inhibitors.

#### e. Hepsin

The gene for hepsin encodes a type II integral membrane protease that has been observed to take part in cell migration and invasion [61-66]. Hepsin is upregulated, which leads to the overexpression of the gene in prostate cancer tumors. Although the lack of detection of hepsin in either urine or serum makes its use as a biomarker difficult, this gene and its protein product has the potential to be utilized in prostate cancer detection [61-65].

### 3.2. Epigenetic Biomarkers

Epigenetic modifications do not involve nucleotide sequence changes and play a critical role in diverse biological processes such as transcription, DNA repair, and differentiation, and their alterations are involved in cancer [67]. Four major components of the epigenetic machinery are DNA methylation, histone modifications, chromatin compactation and relaxation, and miRNA (and non-coding RNA) expression [68]. A majority of the epigenetic biomarkers are associated with hypermethylation of DNA, especially at promoter sequences [69]. Aberrant DNA methylation is induced at specific promoter CpG islands in contrast to mutations. This hypermethylation often leads to repression of the gene, as in the cases of GSTP-1 and DAB2IP. Epigenetic changes in DNA also include chromatin remodeling and hypermethylation. A few examples are discussed below:

#### a. PDLIM4

In prostate cancer cells, both PDLIM4 mRNA and protein expression are reduced by hypermethylation of the gene. PDLIM4 may act as a tumor suppressor in prostate cancer by controlling cell proliferation and also may predict recurrence. When hypermethylated, this gene can be used as a biomarker in detecting cancer and predicting its recurrence [42]. Regular PCR-based methylation analysis is applied to measure hypermethylation and RTPCR to measure gene expression.

#### b. GSTP-1

Hypermethylation of the GSTP-1 gene leads to the loss of expression of this gene, which has an important function in cells [44]. GSTP-1 encodes a detoxifying enzyme that defends cells against free radical damage to DNA and cancer initiation. Suppression of this gene following hypermethylation may lead to damaged DNA or to a greater likelihood of developing prostate cancer. GSTP-1 may be used as a screening method for prostate cancer detection and is a biomarker for diagnosis [70].

Along with a number of biomarkers that are regulated by epigenetic mechanisms, a common sequence rich in C and G is present near the promoter of genes involved in prostate cancer (and in other cancers as well). CpG islands are portions of DNA with a high number of cytosines and guanines. Hypermethylation in these regions is one of the most common alterations in the carcinoma tissue DNA of the prostate. Because hypermethylation of these CpG islands is not present in normal cells, CpG hypermethylation can be used as a biomarker for the diagnosis and detection of prostate cancer [71,72].

#### c. Micro RNA Profiles

MicroRNAs (miRNAs) are an important class of messenger RNAs (mRNAs) that regulate the expression of multiple genes by post-transcriptional mechanisms [73-75]. miRNA dysregulation has been shown to be involved in diverse physiological processes, development, differentiation, and apoptosis. More than 1,000 types of miRNAs have been reported to date. The role of miRNAs in prostate cancers has been investigated recently in several studies and may offer novel strategies for the prevention, early detection, diagnosis, and treatment of these diseases [76-79]. Investigators used prostate tissue from patients and adjacent normal tissue from the same patient to isolate prostate cancer-specific miRNAs that could distinguish healthy patients from cancer patients. These prostate cancer-specific miRNAs include MIR26A, MIR30D, MIR29A, MIR126, MIR195, MIR145, MIR205, MIR221/225, and MIR342-3P. Attempts are being made to correlate Gleason Score with the expression of different miRNAs [42-44,46]. The role of MIR128 works as a negative regulator of proteomic profiling in prostate cancer and its implication in cell invasion have been demonstrated recently by Khan *et al.* [80]. In these experiments, 15 prostate-derived tissues that included five each from adjacent benign prostate, clinically localized prostate cancer, and metastatic disease from distant sites were used. Such studies should be conducted in large number of samples.

#### d. Polycomb Group Proteins

Polycomb group (PcG) proteins play a role in repressing homeotic genes, which are responsible for the development of the body plan. EZH2, a polycomb protein, is overexpressed in prostate cancer development. PcG proteins are parts of epigenetic systems, and EZH2 in particular is a histone methyltransferase that interacts with DNA methyltransferases. The addition of methyl groups leads to the transcriptional repression of polycomb complexes. Alterations in polycomb complexes serve as biomarkers for prostate cancer progression [81,82].

#### e. DAB2IP

Disabled homolog 2-interacting protein (DAB2IP) is a Ras GTPase-activating protein that serves as a tumor suppressor. The gene that encodes this protein is downregulated in prostate cancer due to altered methylation patterns in the promoter region of this gene. This methylation leads to transcriptional silencing and also may be responsible for the progression of cancer. DAB2IP can be used as a biomarker for diagnosis and can be considered either a genetic or epigenetic biomarker [25].

Few other epigenetic markers include pITX2 (hypermethylation indicated prostate cancer recurrence) [83], sprout 1 [84], PMEPA1 [85], EFEMP1 [86] and PTGS2 [87]. Genome-wide methylation analysis of prostate cancer tissues has also resulted in some new epigenetic markers [88]. In contrast to genomic alterations, epigenetic alterations can be reversed. Reactivation of tumor-suppressor genes by demethylating agents and histone deacetylase inhibitors could be a potential treatment option for patients with advanced prostate cancer.

### 3.3. Proteomic Biomarkers

Proteomics is the study of proteins and in the case of prostate cancer certain proteins serve as effective biomarkers. The power of serum protein profiling in distinguishing prostate cancer from healthy individuals has been demonstrated previously [89]. Among these proteomic biomarkers is PSA, which is one of the first fully accepted and possibly one of the most commonly used biomarkers in the clinic.

#### a. PSA

Increased levels of PSA are positively correlated with advanced prostate cancer [38,44]. PSA serves as a biomarker for prostate cancer screening and early detection, but recent research has shown that PSA may not be as strong a biomarker as previously believed. High PSA levels often may be present in non-malignant cases of cancer, leading to overdiagnosis and overtreatment. This antigen also may yield false-positive information; thus the low specificity and sensitivity of PSA may make it difficult to differentiate between benign and aggressive cancers [57,60,90].

#### b. PAP

Human prostatic phosphatase, also known as serum acid phosphatase or PAP, is a biomarker that was discovered in the 1930′s. At that time scientists established PAP as a diagnostic and prognostic biomarker after noticing that prostate cancer patients whose cancer metastasized to bone had high serum levels of PAP [42].

#### c. AMACR

Alpha-methylacyl-CoA racemase, or AMACR, is an isomerase involved in fat metabolism. It functions as a growth promoter in prostate cancer and is overexpressed in prostate cancer tissue. AMACR has been shown to be a specific biomarker for diagnosis, although there are limitations to its use that involve increased levels of the isomerase in benign conditions [57-60].

#### d. GRN-A

Chromogranin-A (GRN-A) is an acidic protein, and its peptides are useful in monitoring the growth of prostate cells. This protein also can be used to monitor the success and effectiveness of cancer treatments. In addition, GRN-A is considered a prognostic biomarker in prostate cancer patients, especially those with advanced cases [42].

#### e. PSMA

Prostate-specific membrane antigen (PSMA) is an integral membrane protein that also functions as an enzyme. Usually, its levels are higher in primary prostate cancer, and they continue to increase with age. PSMA is classified as a prognostic and diagnostic biomarker for prostate cancer; it generally is detected in prostate tissues, cancer cells, and serum [59].

#### f. PSCA

Prostate stem cell antigen (PSCA) is a membrane glycoprotein that is expressed in prostate cancer. Increased expression of PSCA is related to advanced tumor stages in the prostate, and a positive correlation between PSCA and prostate cancer risk exists. PSCA has been associated with signal transduction in cancer cells, and this glycoprotein also may play a role in prostate cancer progression. Although more research is needed to establish PSCA as an effective biomarker for prostate cancer, it has been shown to be an effective therapeutic target [57].

#### g. EPCA

Early prostate cancer antigen (EPCA) is a nuclear matrix protein that is associated with nuclear transformations in the early development of prostate cancer. EPCA also is found in prostate cancer precursor lesions, and its expression is higher in prostate cancer tissue than in noncancerous cells. As a biomarker, EPCA has diagnostic value and can be detected in the serum of patients [39].

#### h. B7-H3

B7-H3 is an immune molecule that has the potential to shield cancers from the immune system and also can slowdown or stop cancer growth. Expression of this protein has been associated with the development of prostate cancer, however. B7-H3 has the potential to predict recurrence and progression and is used as a diagnostic and prognostic biomarker [42].

#### i. Sarcosine

As an amino acid derivative, sarcosine is a metabolite that influences the malignant growth of benign prostate cancer cells. It is associated with increased cancer cell invasion and cancer aggressiveness. Sarcosine may be used as a biomarker for diagnosis, especially for aggressive prostate cancer [42].

#### j. Cav-1

Caveolin-1 (Cav-1) is an integral membrane protein that is overexpressed in prostate cancer cells. This protein is secreted by cancer cells in the prostate, and functions in the regulation of signaling pathways as well as other intracellular processes. Cav-1 has been observed to be upregulated in metastatic cancers and is related to disease progression. It is used as a prognostic biomarker in prostate cancer [42].

#### k. Ki-67

Antigen Ki-67 is associated with cell proliferation, distant metastasis, and survival in patients with prostate cancer. It serves as both a prognostic and predictive biomarker, especially for men with low-grade and low-stage prostate cancer. Additional studies are being conducted to determine the potential of Ki-67 as a biomarker [42].

#### l. HK2

Human Kallikrein 2 (HK2) is a serine protease with a gene sequence that is similar to that of PSA HK2 is androgen-dependent and is produced in the prostate. Increased concentrations of HK2 in the blood are associated with an aggressive type of prostate cancer, and HK2 typically is overexpressed in prostate cancer tissue. HK2 has been established as a prognostic biomarker for advanced disease and is predictive of advanced and recurrent disease in patients [59].

#### m. PSMA

Prostate-specific membrane antigen (PSMA) is a protein that is embedded in the cell membrane of epithelial cells in the prostate. PSMA expression is much higher in cancerous prostate tissue than in the normal tissue, and it is used to detect prostate cancer in tissue as well as metastasis. PSMA is not fully accepted as an effective biomarker, however [91,92].

#### n. Katanin p60

Katanin p60, a microtubule protein, has been found to be overexpressed in prostate cancer progression and metastasis [18].

### 3.4. Other Biomarkers

#### a. Serum Calcium

Researchers have identified an association between high levels of calcium in serum and the risk of prostate cancer. Prostate cancer cells inherently express calcium-sensing G protein-coupled receptors, but high levels of calcium in serum promote the growth and metastasis of prostate cancer. Calcium levels can be used as a biomarker in screening for or diagnosing prostate cancer [93].

#### b. Vitamin D

Szendroi *et al* [94] demonstrated that vitamin D receptor, estrogen receptor alpha, and calcium sensing receptor genetic polymorphisms had a significant association with the risk of prostate cancer. In another cohort study, Choo *et al*. [95] examined serum 25(OH)-vitamin D levels in patients with nonmetastatic prostate cancer and observed that vitamin D insufficiency was prevalent among these patients. One study of vitamin D showed that low levels of vitamin D are related to an increased risk of developing prostate cancer [96-98]. Low levels of vitamin D may be correlated with more aggressive prostate cancer and with higher cancer incidence and mortality rates [99]. In addition, the researchers in this study determined that obesity is inversely related to prostate cancer mortality; this may be due to the fact that those who are obese also may have insufficient levels of vitamin D. Vitamin D may be both a predictive and diagnostic biomarker for prostate cancer. Halt *et al.* [96] discovered that genetic variations in vitamin D pathway genes were altered both risk of recurrence/progression and prostate cancer specific mortality [96]. However, Barnett and Beer [100] have expressed their views that clinical data have not demonstrated yet any link between vitamin D and prostate cancer. Vitamin D levels may influence incidence rates of other cancers also such as breast cancer, colon cancer, esophageal cancer, and hepatocellular carcinoma.

#### c. Exosomes

Exosomes are vesicles that contain mRNA and microRNA. Generally, the presence of gene fusion rearrangements, as in the case of TMPRSS2-ERG, can be detected in exosomes found in the urine of patients. Exosomes are noninvasive tumor markers, which makes them ideal biomarkers for use in the diagnosis and monitoring of monitoring prostate cancer [42,93].

Biomarkers described above have the potential to be used in epidemiologic studies. In the following section we discuss about two additional topics: over-treatment in prostate cancer and low level biomarkers in prostate cancer.

Prostate cancer over-treatment is a problem which needs further research. Current standard of care is not ideal because it involves either active surveillance or radical therapy. Lecornet *et al.* [101] have suggested focal approach to avoid over and under treatment of prostate cancer and physicians should consider improving accuracy for cancer localization by multi-parameter MRI and new biopsy strategies (transperineal mapping biopsies), ablate modalities (cryotherapy), high intensity focused ultrasound, photodynamic therapy and radio-interstitial tumor ablation. This approach will also reduce psychological morbidity as a result of anxiety and side-effects due to repeated biopsies. Another group has different suggestions to avoid over-diagnosis and over-treatment of prostate cancer [102]. Based on results from two trials they suggested that coordinating screening based on PSA testing should be on hold until a more specific marker for aggressive disease than PSA levels become available. Mohan *et al.* [103] have proposed guidelines to avoid over-treatment of localized prostate cancer. In a separate study, adequate knowledge of prostate cancer levels and realistic perception of the active surveillance strategy in patients with early prostate cancer was observed [104].

Decreased levels of few biomarkers have been observed with progression of prostate cancer which has tremendous clinical application [105]. A few examples include specific glycans, especially F1, F2, and F3 subforms with higher levels of sialic acid than the F4 subform [106], PTEN [107], CXC receptor [108] and zinc [109].

One additional topic which is worth mentioning to cover prostate cancer epidemiology is active surveillance. The concept of active surveillance, or watchful waiting, is a viable option for men who decide not to undergo immediate surgery or radiation therapy. During active surveillance, prostate cancer is carefully monitored for signs of progression. A PSA blood test and digital rectal exam (DRE) are usually administered periodically along with a repeat biopsy of the prostate at one year and then at specific intervals thereafter. Current best estimates indicate that many more men are treated for prostate cancer aggressively than is likely necessary to save a life from the disease. The challenge has been to identify those men who do not need immediate therapy, which is usually decided based on age, and cancer factors like the PSA, stage, amount of cancer in the biopsy, and Gleason grade. Active surveillance might also be a good choice for older men with limited life expectancy. In addition, if a man is currently battling other serious disorders or diseases, such as heart disease, long-standing high blood pressure, or poorly controlled diabetes, his doctors might feel it is in his best interest to hold off on therapy and avoid its potential complications.

## 4. Research Gaps in Prostate Cancer Epidemiology and Future Prospects

Although previous and ongoing research has vastly improved our knowledge of prostate cancer, gaps still exist in prostate cancer epidemiology research. A better understanding of the molecular basis for the development and progression of prostate cancer is needed [71,72]. The fact that the etiology of prostate cancer is uncertain provides additional setbacks and halts the search for preventative measures that could benefit for potential patients. Prostate cancer detection and screening methods also have proven to be less efficient than previously believed. Testing and screening using PSA, one of the most common and earliest adopted biomarker, has been found to be inaccurate, because the protein lacks sensitivity and specificity. Yocum *et al* [110] has emphasized that the measurement of serum PSA suffers from lack of specificity and its inability to distinguish clinical cases in which current treatment measures would be successful. PSA levels can increase as a result of noncancerous conditions such as benign prostatic hyperplasia (BPH), which raises questions about PSA's reliability as a biomarker [38,58]. PSA thresholds for prostate cancer detection also have been shown to be invalid, because these threshold levels may vary from patient to patient [60]. The positive predictive value of PSA is about 10% in men with serum PSA levels of less than 4 ng/mL. Although most patients with PSA levels of less than 10 ng/mL have early stage disease, more than 50% of patients with PSA levels of more than 10 ng/mL are found to have advanced disease [38,89]. Therefore, a low cut off PSA value should be used for early detection of prostate cancer to eliminate false-positive results.

PSA's limitations point to the need for a biomarker that can better distinguish between benign and malignant cancers. The search for biomarkers has not been successful, because the effectiveness of many potential biomarkers has not yet been confirmed. This may be because no standard procedure exists for evaluating and validating biomarkers and/or because the methods currently utilized for this purpose are expensive and time-consuming [42,44,58]. Many of the problems experienced with PSA, such as inadequate sensitivity and specificity, apply to other potential biomarkers as well, and no effective biomarkers have been approved for prostate cancer to date [59]. Currently epigenetic biomarkers are limited to methylation; no effective biomarker, either alone or in combination with other markers have been identified for cancer detection and progression [72]. Furthermore, researchers are not aware of the extent of epigenetic changes in prostate cancer [22].

From a larger perspective, increased focus should be placed on researching prostate cancer prevalence in populations. Despite suggested estimates, the actual prevalence of prostate cancer in the general population is unknown [110]. In addition, many social disparities exist in research. Understanding the mechanisms behind increased prostate cancer in African American men and why incidence and mortality rates are higher in African American men than in other population groups is an example of such a challenge. Research also has not produced an adequate understanding of prostate cancer in men of other cultural backgrounds, including Asian men, who have the lowest prostate cancer incidence and mortality rates.

Management of prostate cancer is recognized as a key medical problem. Whether more research should be focused on circulating tumor cells is a topic of debate. Such studies may provide new insight into the biology of this complex disease and significant implications for the clinical management of patients. Prostate cancer has high rate of recurrence after therapy. It is estimated that more than 25% of all prostate cancer patients will develop local or distant recurrence within few years of initial curative-intended therapy. Half of these patients may need secondary therapy. CTCs may provide tools to follow up treatment response and progression of disease. Although surgery and chemotherapy can remove primary tumor, it has been observed that few CTCs and DTCs remain which may cause metastasis of prostate cancer. Research is also needed in the area of prostate cancer therapy. In one study where patients were treated with surgery, radiation and hormone therapy, it was observed that HER-2 expression played a significant role in androgen resistance and helps prostate cancer prognosis [111]. In this study prostate cancer cells were detected using anti-PSA monoclonal antibodies. Such studies emphasize the importance of characterizing the clinical state of the patient, especially the information about prior hormone exposure.

In the future, additional prostate cancer biomarkers should be validated clinically so that they can be used in screening, detecting, diagnosing, and determining prognosis and survival outcome. Furthermore, interaction between public and private institutions is needed to bring new biomarkers to the clinic. Granting agencies should also place more emphasis on developing biomarkers for medications already in use for prostate cancer. A focus on quantifiable biomarkers of signaling pathways or drugs could increase the applicability of biomarkers; thus increasing the potential for return of investments by sponsoring companies. Pathway biomarkers could also help identify new drug targets and streamline the drug development process. Furthermore, a deeper understanding of the relevance of multiple biomarkers for prostate cancer will be essential to efficiently diagnosing this cancer and directing patients towards medications that are likely to be beneficial, based on the molecular profiles of genes and proteins in the patients being treated. Molecular approaches to diseases should bring reduced complications of treatment and dramatically improved response rates. Economy of burden from prostate cancer is also an area of research. Variation in costs in different countries should be determined. Variation of costs may show variation attributed to difference in incidence and management practices. Factors contributing per patient costs are cancer stage at diagnosis, survival, and choice of treatment. Although mortality rate is declining in most countries, costs are expected to rise due to increased diagnosis, diagnosis at an early stage, and increased survival. Therefore, new strategies should be identified and implemented to increase the efficiency of healthcare provision which will reduce the economic burden of prostate cancer.

## 5. Conclusions

Prostate cancer is a significant public health threat worldwide, particularly in countries where men have life expectancies long enough to clinically manifest this cancer. Prostate cancer epidemiology knowledge has increased tremendously and studies based on profiling of the genome, proteome, and epigenome has provided potential biomarkers and therapeutic targets which will contribute in reducing the burden of this disease. Several elements regarding the diagnosis, prognosis, and management of prostate cancer patients remain enigmatic.

## Figures and Tables

**Table 1.** Different kinds of biomarkers of prostate cancer.

**Biomarkers**	**Description**	**References**
**Genetic**		
TMPRSS2-ERG gene fusion	Gene fusion due to translocation on chromosome 21 of oncogenes drives cell proliferation in prostate cancer (PCa) and tumor progression	[22,27,34-36]
PCA3	Gene encodes prostate-specific noncoding mRNA; antigen can enhance diagnosis of prostate cancer stage, grading, and aggressiveness when detected in urine	[27,34-36]
GOLPH2	GOLPH2 gene encodes a Golgi membrane antigen and is upregulated in 90% of cases, leading to overexpression of the antigen in prostate cancer	[27]
PIM1	Gene encodes a protein kinase; significant PIM1 expression can be found in advanced prostate cancer cases	[38]
Hepsin	Gene encodes a type II integral membrane protease; upregulated in prostate cancer, leading to overexpression of the gene in tumors	[34,35,39]
NKX3A	Encodes a transcription factor that functions in prostate epithelial development; losses in a region containing this gene may lead to prostate cancer development and progression	[22]
PTEN	Loss of function (by allelic loss or mutation) at this tumor suppressor in advanced stages	[22]
RB1	Loss of function (by allelic loss or mutation) at this tumor suppressor in advanced stages	[22]
TP53	Loss of function (by allelic loss or mutation) at this tumor suppressor gene; found in advanced stages of prostate cancer	[22]
**Epigenetic**		
PDLIM4	Hypermethylation leads to reduced PDLIM4 mRNA and protein expression in prostate cancer cells and may be useful in detecting prostate cancer tumorigenesis	[27]
GSTP-1(Gluthione S-transferase P1)	Hypermethylation leads to the loss of expression of GSTP-1, potentially leading to damaged DNA and greater likelihood for prostate cancer development	[27]
CpG islands	Hypermethylation in these regions leads to disruption of the functioning of various genes involved in prostate cancer progression and development and can function in prostate cancer detection; present in multiple cancers	[40,41]
Polycomb components (PcG proteins)	Chromatin modifications, varied composition, and overexpression of polycomb complexes may be indicative of prostate cancer progression	[22,50]
RASSF1A, RARB2, APC, GSTP1 or GSTP1, APC, MDR1	Combined hypermethylation assays for these genes can assist in discriminating between benign alterations and cancerous alterations in the prostate	[22]
ASC/TMS1 (PYCARD)	Gene encodes an immune response regulator, hypermethylation of this gene is found in 40% of cases	[22]
EPB41L3	Gene encodes a cortical cytoskeleton protein, hypermethylation of this gene is found in 70% of prostate cancer cases	[22]
RASSF1A	Hypermethylation in the promoter of this gene is indicative of benign regions in the prostate; a patchy pattern of hypermethylation of this gene promoter is indicative of carcinomas	[22]
DLC1	Methylation of this gene leads to gene repression and increases in prostates of older men; this gene is a biomarker for prostate cancer development in its early stages	[22]
LINE-1 retrotransposons	Hypomethylation of these sequences occurs in metastatic cases indicating prostate cancer development; these retrotransposons are hypermethylated in normal conditions	[22]
CDKN1C	Hypermethylation resulting in inactivation of gene in prostate cancer	[22]
IGF2	Loss of differential methylation pattern associated with loss of imprinting, which appears to set in the aging prostate before manifest carcinomas; IGF2 is a preneoplastic methylation change in aging prostate	[22]
H3K4	Increased dimethylation at lysine residue correlates with poor prognosis of prostate cancer	[22]
H3K18	Increased acetylation activation marker, correlates with poor prognosis	[22]
JMJD3	A demethylase that is overexpressed in metastic prostate cancer	[22]
HDAC1	A histone deacetylase that is found in prostate cancer, harbor TMPRSS2-ERG fusion	[22]
TNFSR10D/DCR2	Encode for preapoptotic receptors DR4 and DR5, mostly down-regulated in prostate cancer, subject to significant hypermethylation	[22]
RNASEL	Hypomethylation results in inactivation; candidate for hereditary prostate cancer gene	[22]
**Proteomic**		
PSA (Prostate-specific antigen)	Antigen, Can be used in disease detection, identifying recurring disease after treatment, levels at diagnosis and more advanced stages	[27]
PAP or AP (Human prostatic acid phosphatase)	Serum biomarker for prostate cancer, high levels of PAP activity in places (bone) where prostate cancer metastasized, high levels in serum, diagnosing metastatic carcinoma of prostate, also a biomarker for progression and reaction to androgen deprivation therapy	[27]
AMACR	Enzyme involved in fat metabolism and is a growth promoter in prostate cancer, is highly specific biomarker used for diagnosis	[27]
GRN-A/CGA (Chromogranin A)	Acidic protein in all neuroendocrine cells, diagnostic and prognostic values	[27]
PSMA	Integral membrane protein with enzymatic properties, used in prostate cancer detection, levels increase in primary prostate cancer and metastatic disease	[54]
PSCA (Prostate Stem Cell Antigen)	Membrane glycoprotein expressed in prostate, prostate cancer detection, indicates more advanced tumor stage with increased expression	[27]
EPCA (Early Prostate Cancer Antigen)	Nuclear matrix protein, linked with nuclear transformations that occur in early prostate cancer, diagnostic	[27]
B7-H3	Immune molecule that participates in development of prostate cancer, helps predict recurrence and progression, may be used as diagnostic/prognostic marker, its expression associated with aggressive disease and short survival	[27]
Sarcosine	Amino acid derivative of glycine, promotes prostate cancer cells toward invasion and aggressiveness, indicator of malignancy	[27]
Caveolin-1 (Cav-1)	Integral membrane protein, overexpressed in prostate cancer cells and associated with progression, low levels related to high Gleason score, prognostic marker	[27]
Ki-67	Cell-proliferation associated marker, protein, can provide predictive and prognostic information	[27]
HK2	A serine protease with structural homology with PSA; marker of disease aggression	[36]
Prostate specific membrane antigen	Embedded in cell membrane of epithelial cells of prostate; marker of metastasis	[54]
DAB2IP (DAB2 interacting protein)	Ras GTPase-activating protein, tumor suppressor, functions in progression of prostate cancer, biomarker for diagnosis	[27]
TRAIL (TNF-related apoptosis-inducing ligand)	Deals with apoptosis, loss of responsiveness to this is characteristic of progressive prostate cancer, TRAIL receptors encoded by 4 TNFRSF10 genes	[22]
